# Impact of Surgical Margin Control in Index Tumors on Prognosis After Radical Prostatectomy: A Focus on Zonal Origin

**DOI:** 10.3390/curroncol32080445

**Published:** 2025-08-07

**Authors:** Jun Akatsuka, Yoshihiko Ogata, Kotaro Obayashi, Mami Takadate, Shunsuke Ikuma, Hiroya Hasegawa, Hikaru Mikami, Hayato Takeda, Yuki Endo, Takayuki Takahashi, Kaori Ono, Yuka Toyama, Yoichiro Yamamoto, Go Kimura, Yukihiro Kondo

**Affiliations:** 1 Department of Urology, Nippon Medical School, 1-1-5 Sendagi, Bunkyo-ku, Tokyo 113-8603, Japan; 2 Department of Urology, Nippon Medical School Tama Nagayama Hospital, Tokyo 206-8512, Japan; 3 Pathology Informatics Team, RIKEN Center for Advanced Intelligence Project, Tokyo 103-0027, Japan; 4 Mathematical Intelligence for Medicine, Graduate School of Medicine, Tohoku University, Sendai 980-8575, Japan

**Keywords:** prostatic neoplasms, index tumor, surgical margin, prostatectomy

## Abstract

We investigated the clinical significance of positive surgical margins (PSMs) in index tumors following radical prostatectomy (RP), with particular attention to the tumor’s zonal origin. Among 1148 patients with localized prostate cancer who underwent RPs, 973 were included after excluding those who received perioperative therapy or had incomplete data. Index tumors were categorized by zonal origin: transition zone, peripheral zone, or central zone (CZ). Overall, PSMs were observed in 26.4% of index tumors. Although CZ index tumors were relatively uncommon (6.5%), they exhibited the highest PSM rate (42.9%) and showed the most aggressive pathological features. The 5-year biochemical recurrence (BCR)-free survival rate was significantly lower in patients with PSMs in index tumors than in those with negative surgical margins (45.6% vs. 86.8%, *p* < 0.0001). Notably, patients with PSMs in CZ index tumors had the worst outcomes, with a 5-year BCR-free survival rate of 22.0%. Multivariate analysis identified PSMs in index tumors as an independent predictor of BCR (HR: 3.4; 95% CI: 2.5–4.5), with a similar trend observed in early recurrence. These findings highlight the prognostic significance of PSMs in index tumors during RP, especially in CZ tumors, and emphasize the importance of securing local control in these cases.

## Simple Summary

We investigated the clinical relevance of positive surgical margins in index tumors following radical prostatectomy, with attention to the tumor’s anatomical location. Among 973 patients, positive margins in index tumors were identified in 26.4%. Although tumors located in the central zone of the prostate were relatively uncommon, they had the highest rate of positive margins (42.9%) and were associated with more aggressive pathological features. After surgery, patients were monitored for a mean duration of 75.0 months, during which recurrence occurred in 216 of 973 patients (22.2%). Patients with positive margins had markedly lower 5-year recurrence-free survival rates compared to those with negative margins (45.6% vs. 86.8%), with the worst outcomes observed in central zone tumors (22.0%). The presence of positive margins in index tumors was significantly associated with cancer recurrence. These findings highlight the importance of achieving negative margins, particularly in tumors originating from the central zone.

## 1. Introduction

Radical prostatectomy (RP) remains the most widely used curative approach for localized prostate cancer, offering the potential for complete tumor removal and long-term disease control [1]. However, positive surgical margins (PSMs), defined as the presence of tumor cells at the surgical cut surface, occur in approximately 6–38% of cases [2,3]. To date, investigations into the significance of PSMs have largely concentrated on their pathological characteristics and associated surgical techniques [3,4]. Among these, the location of PSMs has received considerable attention [5]. PSMs at the anterior, posterior, bladder neck, posterolateral, apical, and soft tissue margins are associated with a higher risk of biochemical recurrence (BCR) than negative surgical margins (NSMs) [6,7,8]. Controlling PSMs is an imperative factor influencing oncological outcomes following RP and remains a major concern for patients and urological surgeons [2,5].

The multifocal nature of prostate cancer is well recognized, with each tumor nodule potentially exhibiting distinct pathological and molecular characteristics [9,10,11]. Recent studies have demonstrated that these nodules often originate from genetically distinct clonal populations, contributing to the heterogeneous and complex clinical behaviors of the disease [12,13]. Increasing evidence suggests that the index tumor—the largest and most biologically aggressive tumor focus—is the main driver of disease progression and metastasis [14,15,16]. Based on these concepts, accurate identification and appropriate management of the index tumor are critical components for optimizing oncological outcomes [17,18].

The zonal origin of prostate cancer refers to the anatomical regions within the prostate, namely the transition zone (TZ), peripheral zone (PZ), and central zone (CZ), where the tumor arises based on the McNeal zonal anatomy classification [19]. Although previous studies have explored the clinicopathological relevance of the index tumor’s zonal origin [19,20,21,22], the relationship between the index tumor location and PSMs has not been well characterized. This study evaluated the incidence of PSMs in index tumors and their prognostic significance for BCR following RP in a Japanese single-institution cohort, with particular attention paid to the zonal origin of the tumor. These analyses aimed to generate insights that may contribute to the optimization of surgical strategies for prostate cancer and support individualized treatment approaches.

## 2. Materials and Methods

### 2.1. Study Population

This retrospective, single-center study included patients with localized prostate cancer who underwent RPs at Nippon Medical School Hospital (NMSH) between 2000 and 2019. Patients were identified through a review of institutional electronic medical records. Patients who received perioperative therapies, such as androgen deprivation therapy or radiation therapy, were excluded. Only patients with adequate postoperative clinical follow-up were included in the final analysis. The study was approved by the Institutional Review Board (IRB) of NMSH (Approval Number: 28-11-663) and was conducted in accordance with the principles of the Declaration of Helsinki. The requirement for written informed consent was waived due to the retrospective nature of the study; however, all eligible patients were given the opportunity to opt out via the NMSH IRB website.

### 2.2. Histopathological Examination

All histopathological evaluations in this study were conducted by a single pathologist with expertise in genitourinary pathology, using the 2014 International Society of Urological Pathology (ISUP) grading system [23]. Whole-mount prostatectomy specimens were serially sectioned at 3–5 mm intervals from the apex to the bladder neck, perpendicular to the rectal surface. Each section was stained with hematoxylin and eosin, and the slides were meticulously reviewed to assess tumor location, extent, and morphology. Tumor foci were identified and marked on each slide. The index tumor was defined as the largest tumor nodule among all foci, following previously reported studies [10,11]. This approach was adopted because tumor size on whole-mount sections is relatively easy to evaluate and has been established to have prognostic significance. Furthermore, the index tumor was classified as arising from the PZ, TZ, or CZ, based on McNeal’s zonal anatomical classification system [24,25]. The classification was determined by the anatomical location of the majority of the tumor, considering factors such as laterality (left or right), anteroposterior position (anterior or posterior), longitudinal level (base, mid-gland, or apex), and proximity to surrounding structures, including the urethra, seminal vesicles, bladder, and ejaculatory ducts. In cases where the index tumor extended across multiple zones, the zone containing the majority of the tumor volume was designated as the primary site of origin.

### 2.3. Follow-Up and Outcomes

Postoperative serum prostate-specific antigen (PSA) levels were measured within six weeks after surgery and every three months thereafter. BCR was defined as a PSA level > 0.2 ng/mL on two consecutive measurements. If the postoperative PSA level did not fall below 0.2 ng/mL, the date of surgery was considered the date of recurrence.

### 2.4. Descriptive and Comparative Analyses

Clinical variables (age, body mass index [BMI], total prostate volume, preoperative PSA level, clinical T stage, European Association of Urology [EAU] classification, and pathological characteristics [biopsy and RP Gleason grade groups, pathological T stage, nerve-sparing approach and lymph node dissection status, pathological lymph node metastases, surgical margin status, and index tumor location]) were summarized using means with standard deviations or proportions, as appropriate. The patients were first categorized into two groups according to the surgical margin status of the index tumor: (1) index tumor surgical margin-positive (index-PSM) and (2) index tumor surgical margin-negative (index-NSM). The index-NSM group was further divided into two subgroups: (a) patients with PSMs on non-index tumors (other-PSM), and (b) patients with NSMs on all tumor foci (all-NSM). Group comparisons were performed.

### 2.5. Survival and Multivariate Analyses

Continuous variables between the two groups were analyzed using the Mann–Whitney U test, while categorical variables were analyzed using Fisher’s exact test. The BCR-free survival (BCR-FS) rate for the overall cohort, as well as for subgroups stratified by index tumor location (PZ, TZ, and CZ) and EAU risk classification, was estimated using the Kaplan–Meier method. Differences in survival curves between groups were evaluated using the log-rank test. Cox proportional hazards models were used to identify predictors of BCR. In addition, logistic regression analysis was performed to identify predictors of early recurrence, defined as BCR within 1 year after surgery, with a focus on its prognostic significance. Clinically relevant variables and those with significance in the univariate analysis were included in the multivariate models. All statistical analyses were conducted using JMP version 16.1.0 (SAS Institute Inc., Cary, NC, USA), and two-sided *p*-values < 0.05 were considered statistically significant.

## 3. Results

**Figure 1:**
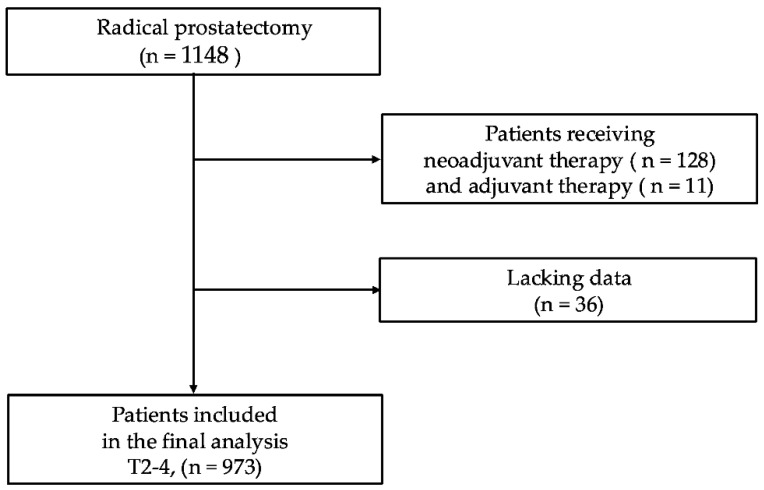
Patient selection flowchart for final analysis.

The study profiles are illustrated in Figure 1. A total of 1148 patients were identified from the institutional medical database. We excluded 128 patients with a history of preoperative adjuvant therapy, 11 who received postoperative adjuvant therapy, and 36 with insufficient data, including those with an undetermined index tumor location. Consequently, 973 patients who underwent RPs at the NMSH were included in the final analysis; all patients were of Asian descent. The clinicopathological characteristics are presented in Table 1. The mean age was 66.8 ± 6.0 years. Among them, 115 patients (11.8%) had preoperative PSA levels of >20 ng/mL, and based on the EAU risk stratification system, the study cohort consisted of 131 (13.5%) low-risk, 440 (45.2%) intermediate-risk, and 402 (41.3%) high-risk cases. Regarding ISUP grading, 344 patients (35.4%) had ISUP grades 4–5 at RP, and 319 (32.8%) had pathological stages pT3-4. Unilateral and bilateral nerve-sparing procedures were performed in 263 (27.0%) and 44 (4.5%) patients, respectively, whereas no nerve-sparing was performed in 666 patients (68.4%). Limited and extended lymph node dissections were performed in 418 (43.0%) and 38 (3.9%) patients, respectively, while 517 patients (53.1%) underwent no lymph node dissections. Pathological lymph node metastases (pN positive) were identified in 14 patients (1.4%). PSMs were observed in 331 patients (34.0%). The zonal origin of the index tumors was the TZ in 316 patients (32.5%), PZ in 594 (61.0%), and CZ in 63 (6.5%).

The clinicopathological characteristics stratified by the surgical margin status of the index tumor are shown in Table 2. Index-PSM was observed in 257 patients (26.4%), whereas index-NSM was noted in 716 patients (73.6%) (other-PSM: 74 patients [7.6%]; all-NSM: 642 patients [66.0%]). In the index-PSM group, the proportion of patients with PSA levels > 20 ng/mL (67 patients, 26.1%), ISUP grades 4–5 at RP (128 patients, 49.8%), and stages pT3–4 (146 patients, 56.8%) were significantly higher than in the index-NSM group (all *p* < 0.0001). The rate of nerve-sparing surgery was found to be significantly lower in the index-PSM group, with 68 cases accounting for 26.5%, compared to the index-NSM group (*p* = 0.04). The clinicopathological characteristics stratified by the location of the index tumors are presented in Table 3. The CZ group showed the highest rate of index-PSM (42.9%). Additionally, the proportions of ISUP grades 4–5 (61.9%), stages pT3–4 (66.7%), and pN positivity (9.5%) were higher in the CZ group than those in other zones, indicating more adverse clinicopathological features.

**Figure 2:**
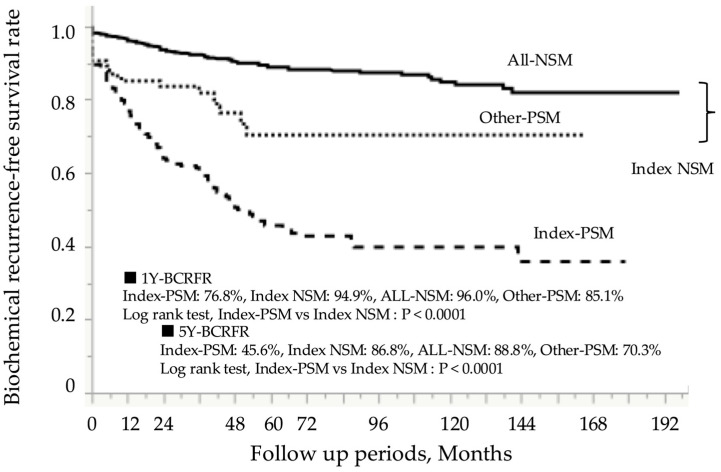
Impact of index tumor PSM on biochemical recurrence-free survival. PSM, positive surgical margin; NSM, negative surgical margin; Index-PSM, index tumor surgical margin-positive; Index-NSM, index tumor surgical margin-negative; Other-PSM, PSM in non-index tumors; All-NSM, NSM in all tumor foci; 1Y-BCRFR, 1-year biochemical recurrence-free survival rate; 5Y-BCRFR, 5-year biochemical recurrence-free survival rate.

**Figure 3:**
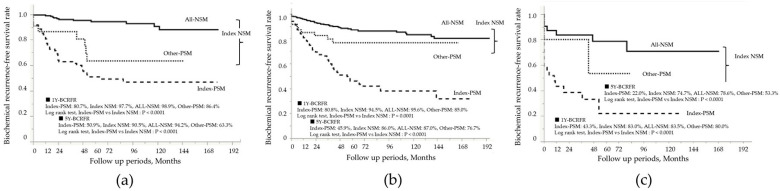
Kaplan–Meier curves stratified by index tumor PSM status in (**a**) transition zone tumors, (**b**) peripheral zone tumors, and (**c**) central zone tumors. PSM, positive surgical margin; NSM, negative surgical margin; Index-PSM, index tumor surgical margin-positive; Index-NSM, index tumor surgical margin-negative; Other-PSM, PSM in non-index tumors; All-NSM, NSM in all tumor foci; 1Y-BCRFR, 1-year biochemical recurrence-free survival rate; 5Y-BCRFR, 5-year biochemical recurrence-free survival rate.

**Figure 4:**
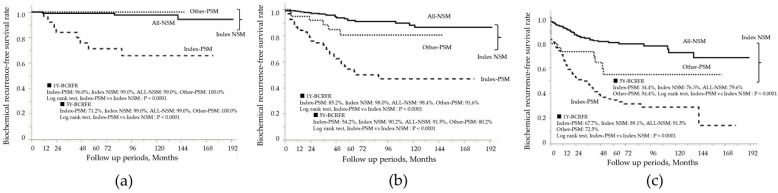
Kaplan–Meier curves stratified by index tumor PSM status in (**a**) the EAU Low-Risk Group, (**b**) the EAU Intermediate-Risk Group, and (**c**) the EAU High-Risk Group. PSM, positive surgical margin; NSM, negative surgical margin; Index-PSM, index tumor surgical margin-positive; Index-NSM, index tumor surgical margin-negative; Other-PSM, PSM in non-index tumors; All-NSM, NSM in all tumor foci; 1Y-BCRFR, 1-year biochemical recurrence-free survival rate; 5Y-BCRFR, 5-year biochemical recurrence-free survival rate; EAU, European Association of Urology.

After RP, patients were monitored for a mean duration of 75.0 ± 49.4 months, during which BCR occurred in 216 of 973 patients (22.2%). Figure 2 presents the Kaplan–Meier curves according to surgical margin status. The 5-year BCR-FS rate in the index-PSM group was 45.6%, which was significantly lower than the 86.8% rate observed in the index-NSM group (log-rank test, *p* < 0.0001). Prognostic analysis based on the index tumor location showed that the 5-year BCR-FS rate in the index-PSM group was 50.9% for TZ, 45.9% for PZ, and 22.0% for CZ, with the CZ group having the worst prognosis (Figure 3a–c). In all zonal groups, the index-PSM group had significantly worse outcomes than the index-NSM group. These trends remained consistent even after stratification by the EAU risk classification (Figure 4a–c). The results of the Cox proportional hazards model for identifying significant predictors of postoperative BCR are shown in Table 4. Multivariate analysis indicated that preoperative PSA levels > 20 ng/mL (hazard ratio [HR] 2.0; 95% confidence interval [CI], 1.3–3.0; *p* = 0.001), ISUP grades 4–5 at RP (HR 3.4; 95% CI, 2.2–5.5; *p* < 0.0001), and pathological stages pT3–4 (HR 1.9; 95% CI, 1.4–2.5; *p* = 0.0001) were all significant predictors of BCR. Additionally, index-PSM emerged as a strong independent prognostic factor for BCR (HR 3.4; 95% CI, 2.5–4.5; *p* < 0.0001). Table 5 presents the univariate and multivariate logistic regression analyses for predicting BCR within 1 year. Index tumor location in the CZ was identified as an independent prognostic factor (odds ratio [OR] 3.7, 95% CI 1.6–8.5; *p* = 0.002). Additionally, the presence of PSM in the index tumor was significantly associated with poorer outcomes (OR 3.1, 95% CI 1.8–5.1; *p* < 0.0001). 

## 4. Discussion

We demonstrated, through whole-mount pathological evaluation of RP specimens, that PSMs of the index tumor served as a significant adverse prognostic factor for BCR, with a similar trend observed in cases of early recurrence. Notably, although index tumors in the CZ group were relatively rare, patients with PSMs showed the worst oncological outcomes.

The control of surgical margins after RP is recognized as a major determinant of oncological outcomes and is a central focus for patients and clinicians [2,5,26]. A systematic review reported that the average PSM rate is approximately 15% (range: 6.5–32%), with stage-specific rates of 9% (range: 4–23%) for pT2, 37% (range: 29–50%) for pT3, and 50% (range: 40–75%) for pT4 disease [2]. Although previous studies have established PSMs as an independent prognostic factor for BCR in patients with prostate cancer treated with RP [6,7,8], the prognostic significance of PSM location remains a point of clinical attention in the context of RP. A recent network meta-analysis demonstrated that the risk of BCR after RP varies according to the PSM location. Among these, anterior margins were associated with the highest recurrence risk, with a reported HR of 2.46 (95% CI, 1.67–3.61), indicating a significantly elevated risk compared to other margin sites [5]. In the largest multicenter Japanese study, PSM location was identified as a significant predictor of BCR, with multifocal and seminal-vesicle-only PSMs associated with significantly worse BCR-FS than apex-, middle-, and bladder-neck-only PSMs [27]. Furthermore, apex-only PSMs, the most common PSM site, were associated with more favorable BCR-FS rates than middle-only or bladder-neck-only PSMs. Although the prognostic impact of PSM locations has been explored, the specific effect of surgical margin status at the index tumor site has not been thoroughly investigated. In this study, we demonstrated that the margin status of the index tumor is a significant predictor of BCR, with a consistent association found in cases of early recurrence.

Previous studies have demonstrated that tumor location is significantly associated with distinct pathological characteristics [20,21]. Among these locations, the CZ is of particular interest because it surrounds the ejaculatory ducts and is anatomically adjacent to the bladder anteriorly and the neurovascular bundles posterolaterally [19]. Cohen et al. analyzed 1767 index tumors and reported that 77% were located in the PZ, 19.7% in the TZ, and 3.3% in the CZ [22]. Their study also demonstrated that prostate cancers arising in the CZ exhibited higher pathological aggressiveness and were more frequently associated with adverse features such as extraprostatic extension and lymphovascular invasion. Previously, we evaluated 621 patients who underwent RPs with NSMs, focusing on the prognostic impact of index tumor location [21]. The 1-year BCR-FS rates for patients with tumors located in the TZ, PZ, and CZ were 99.5%, 95.7%, and 83.3%, respectively. Index tumors in the CZ demonstrated the highest risk of early BCR, and multivariate analysis identified index tumors in the CZ as the most significant independent predictor of early BCR. Based on these findings, we assessed the impact of surgical margin control, with particular attention paid to the index tumor location. Our study demonstrates the importance of meticulous surgical margin control, particularly when the index tumor arises in the CZ, which, although uncommon, is associated with a higher incidence of PSMs and, consequently, worse oncological outcomes. Although this study focused on margin status based on the zonal origin of the index tumor, it did not assess the anatomical location of PSMs in non-index tumors. Future studies investigating the anatomical distribution of PSMs in non-index tumors would be valuable in validating and expanding upon our findings, thereby reinforcing their clinical relevance, with implications for personalized surgical strategies in prostate cancer management.

Index tumors play a pivotal role in disease progression [14,15,16]; thus, accurate pretreatment localization of this lesion is of paramount importance. Recent studies have demonstrated that combining multiparametric magnetic resonance imaging (mpMRI) with fusion-targeted biopsies achieved high diagnostic accuracy, enabling the precise identification of index tumors [28]. Moreover, ^68^Ga prostate-specific membrane antigen positron emission computed tomography has been suggested to offer incremental improvements over mpMRI in the localization of index tumors, highlighting its potential utility as a diagnostic tool for prostate cancer [29]. Furthermore, there is an increasing interest in focal therapy targeting the index tumor, particularly in the context of advanced imaging techniques. The integration of these high-resolution imaging modalities has positioned focal therapy for index tumors as a rational intermediate treatment strategy with the potential to mitigate overtreatment while minimizing the risks associated with disease progression, psychological distress, and treatment-related morbidity [30]. The present analysis, based on histopathological evaluation using whole-mount specimens, underscores the clinical relevance of achieving control of the index tumor in the context of RPs. Accurate localization of the index tumor may be a critical prognostic determinant, even in the context of RPs.

This study had some limitations. First, this was a single-center retrospective study involving only Asian patients, which may limit the generalizability of the results due to potential selection bias [31]. Second, in most cases, pelvic lymph node dissection was limited in extent, resulting in a low rate of histopathologically confirmed lymph node metastasis (1.4%). Adjuvant therapy was given to only 11 of 1148 patients and was excluded from analysis based on clinical judgment, warranting cautious interpretation (Figure 1). Lastly, this study did not assess the association between PSMs and oncological outcomes such as metastatic progression or cancer-specific mortality. Moreover, the relatively short mean follow-up duration of 75.0 months may have limited the ability to evaluate such outcomes. However, in an additional analysis focusing on BCR within 1- year, index PSM remained a strong adverse prognostic factor, further supporting the findings of this study. A strength of this study is that all surgeries and pathology reviews were performed by the same team, ensuring consistency and data reliability. Future multicenter prospective studies with larger cohorts are needed to validate these findings.

## 5. Conclusions

Our study demonstrated that PSMs of the index tumor were a significant adverse prognostic factor for BCR in patients who underwent RP, with a similar trend observed in those who experienced early BCR within 1 year after surgery. Notably, although CZ tumors are rare, they are associated with high PSM rates and the worst prognoses. These findings highlight the need for surgical strategies tailored to the index tumor location, particularly in patients with CZ index tumors, to improve oncological outcomes and guide perioperative prostate cancer management.

## Figures and Tables

**Table 1:** Clinicopathological characteristics of the entire cohort.

Patient in the Entire Cohort: N = 973			
Age, years, average ± SD		66.8	±6.0
BMI, kg/m^2^, average ± SD		23.6	±2.9
TPV, mL, average ± SD		30.7	±15.0
Preoperative PSA, (ng/mL), n, (%)	≤10	592	60.8
	10–20	266	27.3
	>20	115	11.8
cT stage, n, (%)	T1	254	26.1
	T2	634	65.2
	T3–4	85	8.7
Biopsy ISUP grade, n, (%)	1–2	524	53.9
	3	190	19.5
	4–5	259	26.6
EAU risk, n, (%)	Low	131	13.5
	Intermediate	440	45.2
	High	402	41.3
Prostatectomy ISUP grade, n, (%)	1–2	422	43.4
	3	207	21.3
	4–5	344	35.4
pT stage, n, (%)	T2	654	67.2
	T3–4	319	32.8
Nerve sparing surgery, n, (%)	Unilateral/Bilateral	263/44	27.0/4.5
	None	666	68.4
Lymph node dissection, n, (%)	Limited/Extended	418/38	43.0/3.9
	None	517	53.1
pN positive, n, (%)		14	1.4
PSM, n, (%)		331	34.0
Index tumor location, n, (%)	TZ	316	32.5
	PZ	594	61.0
	CZ	63	6.5

SD, standard deviation; BMI, body mass index; TPV, total prostate volume; PSA, prostate-specific antigen; cT, clinical T; EAU, European Association of Urology; ISUP, International Society of Urological Pathology; pT, pathological T; pN, pathological lymph node metastasis; PSM, positive surgical margin; TZ, transition zone; PZ, peripheral zone; CZ, central zone.

**Table 2:** Comparison of patient characteristics based on index tumor surgical margin status.

		Index-PSM (n = 257:26.4%)		Index-NSM (n = 716:73.6%)				Index-PSM vs. Index-NSM
				Other-PSM (n = 74:7.6%)		All-NSM (n = 642:66.0%)		
Age, y, average ± SD		67.4	±5.7	67.3	±5.4	66.5	±6.2	0.15
TPV, mL, average ± SD		30.5	±16.5	30.8	±14.5	30.7	±14.5	0.66
BMI, kg/m^2^, average ± SD		23.7	±3.0	24.3	±2.9	23.5	±2.8	0.90
Preoperative PSA (ng/mL), n, (%)	≤10	117	45.5	42	56.8	433	67.4	<0.0001
	10–20	73	28.4	24	32.4	169	26.3	
	>20	67	26.1	8	10.8	40	6.2	
Prostatectomy ISUP grade, n, (%)	1–2	73	28.4	27	36.5	322	50.2	<0.0001
	3	56	21.8	22	29.7	129	20.1	
	4–5	128	49.8	25	33.8	191	29.8	
pT stage, n, (%)	T2	111	43.2	38	51.4	505	78.7	<0.0001
	T3–4	146	56.8	36	48.6	137	21.3	
Index tumor location, n, (%)	TZ	96	37.4	22	29.7	198	30.8	0.003
	PZ	134	52.1	47	63.5	413	64.3	
	CZ	27	10.5	5	6.8	31	4.8	
Nerve sparing surgery, n, (%)		68	26.5	18	24.3	221	34.4	0.04
Lymph node dissection, n, (%)		155	60.3	42	56.8	259	40.3	<0.0001
pN, n, (%)		9	3.5	3	4.1	2	0.3	0.003

PSM, positive surgical margin; NSM, negative surgical margin; Index-PSM, index tumor surgical margin-positive; Index-NSM, index tumor surgical margin-negative; Other-PSM, PSM in non-index tumors; All-NSM, NSM in all tumor foci; SD, standard deviation; TPV, total prostate volume; BMI, body mass index; PSA, prostate-specific antigen; ISUP, International Society of Urological Pathology; pT, pathological T; TZ, Transition Zone; PZ, Peripheral Zone; CZ, Central Zone; pN, pathological lymph node metastasis.

**Table 3:** Clinicopathological features according to index tumor zonal origin.

		TZ (316: 32.5%)		PZ (594: 61.0%)		CZ (63: 6.5%)		TZ vs. PZ	TZ vs. CZ	PZ vs. CZ
Age, years, average ± SD		66.7	±5.7	66.9	±6.2	66.4	±5.8	0.34	0.70	0.40
BMI, kg/m^2^, average ± SD		23.6	±2.9	23.6	±2.8	24.2	±3.2	0.97	0.17	0.14
Preoperative PSA (ng/mL), n, (%)	≤10	166	52.5	401	67.5	25	39.7	<0.0001	0.03	<0.0001
	10–20	86	27.2	152	25.6	28	44.4			
	>20	64	20.3	41	6.9	10	15.9			
EAU risk classification, n, (%)	Low	33	10.4	94	15.8	4	6.3	0.06	0.01	<0.0001
	Intermediate	148	46.8	276	46.5	16	25.4			
	High	135	42.7	224	37.7	43	68.3			
Prostatectomy ISUP grade, n, (%)	1–2	156	49.4	254	42.8	12	19.0	0.16	<0.0001	<0.001
	3	64	20.3	131	22.1	12	19.0			
	4–5	96	30.1	209	35.2	39	61.9			
pT stage, n, (%)	T2	221	69.9	412	69.4	21	33.3	0.88	<0.0001	<0.001
	T3–4	95	30.1	182	30.6	42	66.7			
Nerve sparing surgery, n, (%)		97	30.7	192	32.3	18	28.6	0.65	0.88	0.57
Lymph node dissection, n, (%)		142	44.9	272	45.8	42	66.7	0.83	0.002	0.002
pN positive, n, (%)		3	0.9	5	0.8	6	9.5	1.0	0.0009	0.0002
Index-PSM, n, (%)		96	30.4	134	22.6	27	42.9	0.01	0.06	0.001

TZ, Transition Zone; PZ, Peripheral Zone; CZ, Central Zone; SD, standard deviation; BMI, body mass index; PSA, prostate-specific antigen; EAU, European Association of Urology; ISUP, International Society of Urological Pathology; pT, pathological T; pN, pathological lymph node metastasis; Index-PSM, index tumor surgical margin-positive.

**Table 4:** Univariate and multivariate Cox regression analyses for predicting biochemical recurrence.

Variable		Univariate HR (95% CI)	*p*-Value	Multivariate HR (95% CI)	*p*-Value
Age, years	≤67	-	-		
	>67	0.9 (0.7–1.1)	0.25		
Preoperative PSA, ng/mL	≤10	-	-	-	-
	10–20	3.1 (2.2–4.2)	<0.0001	2.1 (1.5–3.0)	<0.0001
	>20	5.5 (3.9–7.8)	<0.0001	2.0 (1.3–3.0)	0.001
EAU risk	Low	-	-		
	Intermediate	2.2 (1.2–4.4)	0.01		
	High	6.5 (3.5–12.1)	<0.0001		
Prostatectomy ISUP grade	1–2	-	-	-	-
	3	3.4 (2.3–5.0)	<0.0001	1.9 (1.3–2.9)	0.003
	4–5	8.6 (5.9–13)	<0.0001	3.4 (2.2–5.5)	<0.0001
pT stage	T2	-	-	-	-
	T3-4	4.7 (3.5–6.2)	<0.0001	1.9 (1.4–2.5)	0.0001
Index tumor location	TZ	-	-	-	-
	PZ	-	-	1.5 (1.1–2.0)	0.01
	CZ	2.9 (1.8–4.6)	<0.0001	1.8 (1.1–2.9)	0.02
Index-PSM	Negative	-	-	-	-
	Positive	5.3 (4.0–6.9)	<0.0001	3.4 (2.5–4.5)	<0.0001

HR, hazard ratio; CI, confidence interval; PSA, prostate-specific antigen; EAU, European Association of Urology; ISUP, International Society of Urological Pathology; pT, pathological T; TZ, Transition Zone; PZ, Peripheral Zone; CZ, Central Zone; Index-PSM, index tumor surgical margin-positive.

**Table 5:** Univariate and multivariate logistic regression analyses for predicting biochemical recurrence within 1 year.

Variable		Univariate OR (95% CI)	*p*-Value	Multivariate OR (95% CI)	*p*-Value
Age, years	≤67	-	-		
	>67	0.9 (0.6–1.4)	0.59		
Preoperative PSA, ng/mL	≤10	-	-	-	-
	10–20	4.3 (2.5–7.3)	<0.0001	2.6 (1.4–4.8)	0.002
	>20	9.5 (5.3–17.1)	<0.0001	3.5 (1.7–7.5)	0.0008
EAU risk	Low	-	-		
	Intermediate	-	-		
	High	14.1 (3.4–58.2)	0.0003		
Prostatectomy ISUP grade	1–2	-	-	-	-
	3	5.1 (2.3–11.2)	<0.0001	2.7 (1.1–6.3)	0.03
	4–5	20.3 (9.4–43.6)	<0.0001	6.8 (2.8–17.0)	<0.0001
pT stage	T2	-	-	-	-
	T3–4	6.2 (3.9–9.9)	<0.0001	1.8 (1.1–3.2)	0.03
Index tumor location	TZ	-	-	-	-
	PZ	-	-	-	-
	CZ	6.4 (3.2–12.5)	<0.0001	3.7 (1.6–8.5)	0.002
Index-PSM	Negative	-	-	-	-
	Positive	5.5 (3.5–8.6)	<0.0001	3.1 (1.8–5.1)	<0.0001

OR, odds ratio; CI, confidence interval; PSA, prostate-specific antigen; EAU, European Association of Urology; ISUP, International Society of Urological Pathology; pT, pathological T; TZ, Transition Zone; PZ, Peripheral Zone; CZ, Central Zone; Index-PSM, index tumor surgical margin-positive.

## Data Availability

The datasets presented in this article are not readily available as they are part of an ongoing study and subject to technical or time limitations.

## Abbreviations

The following abbreviations are used in this manuscript:

RP	Radical prostatectomy
PSM	Positive surgical margins
BCR	Biochemical recurrence
NSM	Negative surgical margins
TZ	Transition zone
PZ	Peripheral zone
CZ	Central zone
NMSH	Nippon Medical School Hospital
ISUP	International Society of Urological Pathology
PSA	Prostate-specific antigen
BMI	Body mass index
EAU	European Association of Urology
Index-PSM	Index tumor surgical margin-positive
Index-NSM	Index tumor surgical margin-negative
Other-PSM	PSM in non-index tumors
All-NSM	NSM in all tumor foci
BCR-FS	BCR-free survival
TPV	Total prostate volume
pT	Pathological T
pN	Pathological lymph node metastasis
HR	Hazard ratio
OR	Odds ratio
CI	Confidence interval
mpMRI	Multiparametric magnetic resonance imaging
